# Assessment of farm households’ perception, beliefs and attitude toward climatic risks: A case study of rural Vietnam

**DOI:** 10.1371/journal.pone.0258598

**Published:** 2021-12-28

**Authors:** Huong Nguyen-Thi-Lan, Shah Fahad, Tuan Nguyen-Anh, Huong Tran-Thi-Thu, Chinh Nguyen-Hong, Nguyen To-The

**Affiliations:** 1 VNU University of Economics and Business, Vietnam National University, Hanoi, Vietnam; 2 School of Economics and Management, Leshan Normal University, Leshan, Sichuan, China; 3 Vietnam National University of Agriculture, Hanoi, Vietnam; 4 Academy of Finance, Hanoi, Vietnam; 5 TIMAS, Thang Long University, Hanoi, Vietnam; Neijiang Normal University, CHINA

## Abstract

Vietnam is one the most vulnerable region to climate change and extreme climatic events, such as flash floods and droughts. This present research aims to explore the farm households’ beliefs, risk perception, adaptive attitude and climate change adaptation measures that they currently utilize in their farms to cope with climatic risks. Further, this study analyzed effect of climate change belief, awareness and adaptive attitudes on farmers’ adaptive behavior. By using structured questionnaire, the data from 816 respondents were collected from seven provinces of Vietnam. We used ordinary least squares regression and logistic regression approach to analyze farmers’ belief, perception of climatic change, and risk attitude towards climatic hazards. Results revealed that farmers in the study area are using the most common climatic risk management strategies including applying new technologies, adjustment of the seasonal calendar, and diversification. Findings further revealed that that farm households were mostly concerned about the risk in soil erosion and washout, followed by the stress of prolonging dry season and droughts. The study participants also reported a decrease in precipitation and increase in temperature and frequency and incidence of other extreme climatic events. A positive significant relationship was found between farm management practices and ecological communities. Risk perceptions and attitude toward climate change are essential factors among farm households of northern mountains of Vietnam. Thus, the climate strain linked with the institutional stress and socio-economic has serious insinuations for farm households’ livelihood bases, a universal climate change adaptation scheme is required to endure farmers’ livelihood.

## 1. Introduction

Effect of climate change on agricultural sector is one of the major challenges in the farming sector. The negative impacts of climate change in agriculture sector are expected to outweigh all the benefits it has brought [1]. Several literature studies have confirmed the instability of temperature that contributes to changing of crops respiration requirement that led to change in time of maturity, relieving net growth as well as productivity of the crops.

Climate change is increasingly affecting the daily life and production of people, especially in mountainous areas where most of the population are ethnic minorities [2, 3]. Despite of the initiative to overcome difficulties, the majority of people lack capacity to understand and interpret the climate information, that make them more susceptible [4–6]. In order to access and implement effective adaptation strategies in agriculture, it is essential to understand farm households’ climatic variation beliefs, mitigation and attitudes toward adaptation [7].

Mountainous areas provide an extensive amount of the world’s agricultural production in the terms of economic value. Sensitivity varies according to the crops, cropping pattern, region and agricultural systems of interest. In certain cases, the limits to crop cultivation seem to be closely interrelated to intensities of economic yield. Yield changeability often rises at higher elevation, so climatic variations may result in a larger risk of yield shortage [8]. This makes agriculture particularly more susceptible to the increase of extreme weather events, including abnormal temperature variations, increased flooding and flash flooding and persistent drought due to climate change [1]. For agricultural production in particular, rises in temperatures during precarious reproductive intervals are anticipated to reduce yields. Secondary impacts of global climatic variations are hard to forecast locally, so that there is vagueness about the impact on agriculture in other areas in the mountainous areas. A study of Smit & Skinner [9] indicated that vulnerabilities of communities are shaped by elements of exposure, sensitivity and adaptive capacity. Mendelsohn [1] pointed out that, people dominate on ecosystem to carry out agricultural activities, so the vulnerability of agriculture is strongly depended on not only climate change biophysical effects but also on the responses of people to overcome those effects.

To sustain productivity and to face the challenges of climatic changes, farm households required to respond to the effective adaptation approaches. Studies of Mendelsohn [1] and Smit & Skinner [9] showed that financial management, agricultural production practices, technological development and schemes of government such as insurance are the four prime strategies to cope with adaptation of climate change effects. In financial management, farmers can diversify sources of income and implement household financial management. Otherwise, purchase of subsidized crop insurance from government side to lower the risk of financial losses caused by poor yields and natural hazards that may lead to sales of industries are also in concerns of farmers to the adapt the strategies.

While research on farm households’ responses and perception of climate change has been done in several countries such as USA (California), UK, South Australia and America [10, 11]. Some researchers focused on the Vietnam farmers’ perceived risks, the attitudes toward adaptation of climatic changes and risk coping techniques that farm households are using at their farms [12, 13]. Studies of Buys et al. [10] and Higginbotham et al. [11] reported that farm households are not willing to believe in the man-made climate change than typical rural and urban inhabitants in Australia. Similarly, Gramig & Barnard [14] reported that farm households assume the human activities are less contributing in climatic variations in Indiana than America, while 79% households assuming it a natural occurrence. According to Higginbotham et al. [11] only 8% of maize farmers in the Midwest showed agreement with the consensus that climatic variations happen mainly due to man-made activities, as in comparison to the American population as of about 49 percent [15].

Vietnam is a country that is considered to be severely affected by climate change and extreme climate events. In 2016, Vietnam had to deal with many different types of weather climatic changes that happening faster and more extreme than the scenario published in 2012 [16]. The extreme and damaging cold in 2016 has seriously affected agricultural production in 14 northern mountainous provinces [17]. Understanding farmers’ perceptions in the northern mountains of Vietnam is important because of their role in national agricultural production, and their part and susceptibility to global climate change. Surprisingly, studies concerning farmer’s belief and adaptation attitude and its effect to farmers’ adaptation behavior are very limited in mountains area, of Vietnam. To fill the research gap, the present research is anticipated to provide a valuable insight about perception of risks related to climatic variation, adaptation behaviors and current adaptation strategies to climatic variation, and both financial and agronomic attitude. This study further reveals the relationship of beliefs regarding climate change, perception risks, risk management strategies and adaptive attitudes implemented among farm households in the northern mountains.

## 2. Research questions and hypothesis

The “most important bottlenecks” is the term using to describe the risk awareness and adaptive capacity perception to climatic variation in several studies [15, 16]. The process of making adaptive or mitigating decisions is prejudiced by a variety of aspects, in which experiences with climate change impacts are the crucial initial step in a hazard assessment [11]. In other words, when people notice proofs of climate change effects, that lead them to be more anxious about climate risk and adaptation [17].

Following the behavioral approach of Fishbein & Ajzen [18], many studies have proved that adaptation behaviors are driven by not only climate change adaptation viewpoint, but also driven by other perceptual elements [22–24]. In this study we discover association of the adaptive risk attitudes and risk coping techniques being utilized by farm households in the northern mountains.

This paper explores two following research questions:

Question 1: What are the climatic strategies (adaptation strategies) farmers of the northern mountains implementing?

The hypothesis following Question 1 is outlined below:

H_1_: With the widespread powerful of science and technology that is conducive to agricultural production, we expected most farmers to be adapting new technologies in production as an adaptation technique, and some of them to be diversifying in production.
Question 2: What are farm households climate change beliefs, climate change perceptions, awareness and adaptive attitudes that affect their adaptive behavior of farmers?
H_2a_: Farm households who consider occurrences of the climatic variation mainly by man-made activities are more anxious of the climate change effects than the farm households who do not believe in the effects of natural calamities.
H_2b_: Climate change beliefs, perceptions and attitudes towards climatic risks and farm household’s adaptation that effect their execution of climate adaptation techniques.

## 3. Material and methods

### 3.1 Surveyed areas and questionnaire

To check the study hypotheses, we designed a questionnaire survey of 816 randomly selected farmers in 7 provinces in northern mountains of Vietnam specifically mountainous areas: Son La, Lai Chau, Hoa Binh, Ha Giang, Lao Cai, Dien Bien and Cao Bang.

A structured questionnaire was used in this study including questions about farmers’ perception related to various weather threats (droughts, extreme rainfall, landslide, etc.), farmers’ beliefs about the weather changes or irregularities in their agricultural production, timely climate risk coping techniques and so on. In order to determine the farmers’ perception and causes of natural calamities, we modified the question of Arbuckle et al. [19] as they first completed farm survey in the Iowa and then carried out a farm survey in the Midwestern United States. The farmers were asked about their climate change beliefs by selecting one of the four answers. The answers were detail ordered as follow: 1 represents the view that climatic events are occurring, that lead to make changes in environment and take full responsibility for climatic variations, 2 represents the view that climatic events are occurring, and it is mainly caused by man-made activities, 3 represents that the climatic variations are occurring, and it is because of environmental changes and man-made activities, and 4 represents the people who showed agreement that there is no sufficient evidence exists to observe that climate change is occurring or not.

Following studies of Le Dang et al. [13] climate risk perception were measured through climatic variations and its adverse impacts. In this study, we measured risk perception by creating an average response scale with eight items mainly focused on farm households’ concerns about hazards to their production (droughts and flash floods, extreme rainfall etc.). To learn about the risk coping techniques investigation, we built a question by following the studies of Hogan et al. [20] as they conducted study in Australia and Arbuckle et al. [19] conducted survey in America. Respondents were required to select the activities that they have conducted in their farms to cope with weather or climate-related risks. Climatic risk coping strategies such as agronomic and financial were listed, in order to describe the farm households plans for implementation of these techniques. Several identical potential techniques suggested by Hogan et al. [20], some related items (such as implementing measures to protect the land and managing water usage) were added in the questionnaire. Table 1 shows the adaptation techniques utilized in the survey.

**Table 1:** Farmers adaptation strategies.

Strategies (adaptation strategies)	Description
1. Diversifying plants and animals	Practicing production
2. Applying new technology	Practice production and infrastructure
3. Adjust the seasonal calendar	Practicing production
4. Implement measures to protect the land	Production practices: digging ditches, ditch
5. Adjust cultivation techniques	Practicing production
6. Managing water usage	Practicing production
7. Diversify sources of income	Financial
8. Household financial management	Investment or shrinking production scale
9. Consolidating safety for people and property	Financial
10. Others	Practicing production and finance

Source: Household Survey.

The consent of each village representative was obtained prior to partaking in the survey. The current research was approved by the Ethics Committee of Vietnam National University. Before conducting the questionnaire survey, consent was obtained from each study participant. The study participants were also informed about the confidentiality, anonymity of the survey. All the study respondents showed their agreement on the aim of the study.

### 3.2 Data analysis

For analysis of the collected data, SPSS and STATA tools were used. Logistic regression has been used to test binary decisions for specific adoption. Ordinary least squares (OLS) regression was applied to predict the adoption of ten adaptation techniques explaining the important factors such as farmers attitude toward risks, farmers’ belief on climate change, adaptation attitudes and others that set the farmers adaptation behavior. The mean score of all techniques, first (not doing and do not plan to) to the (implement as a long-term or short-term risk coping technique) was estimated for each participant and utilized as the dependent variable in the analysis. By the description of the surveyed data, ordinal statistical model was more suitable to be utilized for analysis of this surveyed data. The other variables (independent variables) considered in this study were farmer risk perception, belief and adaptation attitude of farmers, household head and household’s characteristics such as age, gender, access to services, level of education, and their access to credit.

The farmers’ acceptance about adaptation strategies is described by OLS regression equation as:

$$\text{Pr}\left(\text{farmers}\;\text{adopt}\;\text{adaptive}\;\text{strategies}\right)=\beta_{1}\left(\text{risk}\;\text{perception}\right)+\beta_{2}\left(\text{climate}\;\text{change}\;\text{hurting}\;\text{activities}\right)+\beta_{3}\left(\text{climate}\;\text{belief}\right)+\beta_{4}\left(\text{adaptation}\;\text{attitude}\right)+\beta_{5}\left(\text{education}\;\text{level}\;\text{of}\;\text{household}\;\text{head}\right)+\beta_{6}\left(\text{age}\right)+\beta_{7}\left(\text{gender}\right)+\beta_{8}\left(\text{access}\;\text{services}\right)+\beta_{9}\left(\text{access}\;\text{credit}\right)+\beta_{0}$$

We also examined the factors that explain adoption of specific adaptation techniques. Ordered logistic regression model was used for the analysis of the data specifically for the five techniques mostly selected by farm households. In this study we calculated the odds ratio to quantify the effects of risk perception, belief, attitude to farmers’ particular adaptation decision.

Variables such as age of the participant, gender, and education level, were selected for regression analysis where it was appropriate. Among the participants, 88.5% were male households, 1.3% were female households, additionally 10.2% participants refused to show their gender. Further 42.5 years were calculated as the average participants age. Mostly respondents graduated from high school, a few have had universities education (48.5% and 2.3% respectively). 18.6% participants graduated from primary school, 13.7% graduated from lower secondary schools and up to 10.7% respondents were illiterate.

## 4. Results

### 4.1 Weather and climate risk management strategy

As mentioned above, farmers were asked about their risk management strategies to cope with weather and climate risks, and questions related to farmers risk coping techniques were added by following the studies of Hogan et al. [20], Fahad et al. [21], Sarker et al. [22] and Arbukle et al. [19].

Table 2 presents the responses and the distribution of answers that emphasizes the widespread application of adaptive strategies such as applying new technology, diversifying plants and animals, implementing measures to protect the land, adjusting cultivation techniques, managing water usage. Most farmers are knowledgeable about adaptation strategies but only partially implemented. In other words, they don’t implement them fully as a long-term or short-term risk management strategy. In total, 64.1% responses consider the implementation new technology; 37.9% responses understand about it, yet they only implement partially. Only 26.2% responses implement this strategy as a long-term or short-term risk management strategy. This is in line with H_1_, and it is unsurprising that the comprehensiveness of applying new technology to agricultural production in the Northern Uplands of Vietnam. In addition, diversifying plants and animals, implementing measures to protect the land, adjusting cultivation techniques, managing water usage are adaptive measures that have used widely by farmers in here. 63.2% responses consider the implementation of land protection measures to be applied as a risk management strategy or partial implementation. 62.5% of farmers implemented water usage management in short-term or long-term or partially.

**Table 2:** Climate change risk management strategy of northern mountainous farmers.

Adaptation strategy	Implement as a long-term or short-term risk management strategy (%)	Understanding and partial implementation (%)	Understanding but not implementing (%)	Not implemented but interested (%)	Not implemented and no implementation plan (%)
*1*. Diversifying plants and animals	*23*.*4*	*35*.*9*	*24*.*0*	*9*.*6*	*7*.*1*
+ Planting a variety of crops, rearing a variety of animals	21.8	43.1	17.5	11.3	6.3
+ Use a variety of varieties	21.2	47.8	14.7	9.1	7.2
+ Rotational	19.6	34.9	26.7	9.4	9.3
*2*. Applying new technology	*26*.*2*	*37*.*9*	*16*.*4*	*13*.*5*	*6*.*0*
+ Use new varieties	23.9	37.6	22.2	11.0	5.3
+ Apply new production techniques	18.9	46.9	16.7	10.5	7.0
3. Adjust the seasonal calendar	*14*.*8*	*23*.*6*	*39*.*2*	*12*.*3*	*10*.*2*
+ Sowing or early harvesting	16.8	42.8	18.1	11.0	11.3
+ Shorten the time of a crop	17.5	37.4	21.9	12.5	10.7
4. Implement measures to protect the land	*24*.*6*	*38*.*6*	*17*.*9*	*10*.*0*	*8*.*8*
*5*. Adjust cultivation techniques	*23*.*7*	*30*.*1*	*28*.*1*	*10*.*9*	*7*.*2*
+ Change the time of fertilizing, spraying	26.5	30.7	26.1	8.8	7.8
+ Change irrigation time	26.7	29.2	27.1	9.4	7.6
6. Managing water usage	*24*.*0*	*38*.*5*	*23*.*2*	*9*.*7*	*4*.*7*
+ Buy or build a water tank	30.4	31.1	23.3	9.6	5.6
+ Use water sparingly	31.4	35.8	21.6	6.4	4.9
+ Reuse (like using vegetable washing water to water plants)	21.0	47.4	15.1	10.2	6.4
*7*. Diversify sources of income	*18*.*9*	*33*.*0*	*27*.*9*	*11*.*5*	*8*.*7*
+ Find more non-farm jobs	19.5	30.8	27.7	13.6	8.5
+ Shift from farming to livestock (partially or wholly) and vice versa	13.5	37.1	27.6	13.2	8.6
*8*. Household financial management	*13*.*2*	*31*.*4*	*29*.*0*	*16*.*4*	*9*.*9*
+ Enhance or expand current production scale	12.1	19.5	41.7	14.1	12.5
+ Mobilizing capital to invest in new production	11.0	38.6	23.3	15.6	11.5
+ Saving	15.7	26.1	30.3	13.1	14.8
*9*. Consolidating safety for people and property	*14*.*5*	*24*.*0*	*34*.*3*	*14*.*2*	*13*.*0*
+ relocation or reinforcement of assets	12.0	30.1	28.6	13.2	16.1
+ Planting forests or trees	16.2	27.8	27.7	13.5	14.8
+ View or listen to news about disaster forecasts	28.9	27.1	22.1	10.7	11.3
+ Sell or rent a piece of property	10.0	22.8	32.7	17.5	16.9
*10*. *Others*	*8*.*3*	*21*.*1*	*37*.*3*	*15*.*9*	*17*.*4*
+ Buy crop and livestock insurance	8.1	15.6	37.0	18.9	20.5
+ Exit industry, abandon agriculture	6.6	16.8	38.2	13.0	25.4

Source: Household Survey.

Adjustment of the seasonal calendar was considered as a common agronomic adaptation approach with 14.8% currently adjusting and 39.2% are aware of it but never implemented or do not wish to implement it. Farmers are likely less interested in applying financial management measures to households, buying insurance for crops and livestock, or withdrawing from agriculture. 20.5% do not implement buy crop and livestock insurance and no implementation plan and not consider to apply it.

### 4.2 Risk perception and attitude to adaptation

Risk perception of farm households are listed in Table 3, all the risk perception observed variables are accepted and will be used in subsequent analysis. Table 3 shows that farmers in the northern mountains of Vietnam are most concerned about the increase in soil erosion and washout, followed by the stress of prolonging dry season and drought. The agreement/disagreement levels with the affirmation of farmers attitude towards climate change and adaptation strategies (changing weather-patterns that damages farming operations) are added to gain risk perception of the peril due to climate change as illustrated in Table 4.

**Table 3:** Farmer worry related to weather/climate risks affecting production.

Threat	No concerned (%)	A little concerned (%)	Concerned (%)	Very concerned (%)	Extremely concerned (%)
Increasing flash floods	0.7	2.4	20.8	22.3	16.6
Long dry season and drought	0.2	2.3	16.2	26.6	17.3
Increasing harmful insects	0.3	3.3	19.0	25.6	14.7
The rate of sick plants is high	0.5	2.9	19.4	25.9	14.0
Extreme rain is more frequent	0.5	4.3	20.1	24.3	13.7
Increase the amount of land lost	0.7	7.6	16.2	22.6	15.5
Increase in extreme temperatures	0.2	4.3	18.7	25.4	13.9
Increasing erosion and washing away soil	0.5	3.9	15.8	23.8	18.7

Source: Household Survey.

**Table 4:** Farmers’ attitude towards climate change and adaptation.

	n	Totally disagree (%)	Disagree (%)	Looks like that (%)	Agree (%	Totally agree (%)
Adaptation attitude: Farmers should change their production practices in the household	805	0.4	0.9	8.1	66.1	23.4
Climate change is a government responsibility, not an individual	804	20.0	42	12.1	20.2	4.3
Noticed more climatic events in the farming: In the recent 5 years, experienced more unusual perils in the farming	808	0.6	2.0	15.7	59.2	21.2

Source: Household Survey.

### 4.3 Climatic risks awareness and beliefs

According to H_2a_, it was expected to estimate the relationship between the farm household’s belief level in climatic variation (where, 1 = climatic variation is not occurring, and 2 = climatic variation is occurring and triggered mainly due to human activities) and their perceived climatic risks to their own production. Ordinal correlation method was utilized to test H_2a_. Table 5 coincide the Kendall’s correlation coefficients and risk perception responses with strong agreement expectations, which shows that perceived risk has a significant relationship with higher climate change beliefs due to human activities.

**Table 5:** Perception of risks and beliefs in climate change caused by humans.

	Disagree/ Strongly Disagree	Looks like that	Agree/ Strongly Agree	Kendall’s Correlation with climate change belief
Changes in the weather are damaging my household’s production	2.9	17.5	79.6	0.103**
Changes in the weather are damaging my household’s activities	3.6	22.9	73.5	0.075**
I believe that more extreme weather-related events will occur in the future	3.3	14.7	81.9	0.078**
Climate variation is not a big deal because human ingenuity allows us to adapt to changes	35.4	21.0	43.6	−0.095**

** Correlation is significant (2-tailed) at the 0.01 level.

A large number of farm households showed strong agreement that changes in the weather are damaging their household’s production and activities (79.6% and 73.5%). A significant and positive association in the farm households’ level of climatic variations belief and perception of climate variability in their farms was found with the value (p < 0.05), further (p < 0.05) value explores the beliefs levels in climatic variation and perception of climate variability. Reliability analysis was conducted to know the measurement of farm households’ use of adaptation and risk coping strategies (as shown in Table 1). The alpha coefficient for the ten items were calculated as 0.881, that suggests the items have quite high core reliability. For investigation of climatic variation adaptation, risk perceptions, beliefs of climatic variation and attitudes, OLS regression was utilized, and mean adoption of these adaptation approaches was calculated (as shown in Table 6). The regression analysis showed only 13% variations in the adaptation scores, but one explanatory variable showed higher rate of significance. Education, age, gender and access credit were not significantly associated with farmers’ behaviors to adaptation. On the other hand, access services were positive and significant with farmers’ behaviors to adaptation.

**Table 6:** Farmer’s adaptation strategies, perceived risk, and attitudes.

Independent variable/continuous variables	Coefficient
Scale of risk	0.191***
Changes in weather are damaging household’s production	0.114***
Climate change belief (CC is occurring)	0.031
**Adaptation attitude**	
Individual adaptation attitude: Farmers should change their production practices in the household	0.071*
Climate change is a government job, not mine	0.023
Education	0.016***
Age	0.002
Gender	0.059
Access services	0.085*
Access credit	−0.022
R^2^ = 0.1259	

*** p<0.01,

** p<0.05,

* p<0.1.

We further investigated the factors that explain the adoption of existing adaptation approaches. Ordered logistic regression was utilized for the main five strategies; diversifying plants and animals; applying new technology; implementing measures to protect the land; adjust cultivation techniques and managing water usage. These responses were classified into five categories; not implemented and no implementation plan (referred as 1); not implemented but interested (referred as 2); understanding but not implementing (referred as 3); understanding and partial implementation (referred as 4) and implement as a long-term or short-term risk management approach (referred as 5). Results of the ordered logistic regression are shown in Table 7.

**Table 7:** Respondents’ specific adaptation strategies.

Independent Variable/continuous variables	Diversifying plants and animals Odds Ratio	Applying new technology Odds Ratio	Implement measures to protect the land Odds Ratio	Adjust cultivation techniques Odds Ratio	Managing water usage Odds Ratio
Risk scale	0.874*	0.619***	0.676***	0.671***	0.587***
Changing weather are damaging household’s production	1.320***	0.770***	0.820***	0.825***	0.816**
Climate change (CC) belief					
*Inadequate evidence to know either CC is occurring or not*	1.029	1.244**	1.202***	1.126*	0.942
*CC is occurring*, *caused mostly by environmental changes*	1.026	0.882**	1.047	0.954	1.038
*CC is occurring*, *caused natural changes/human activities*	0.905	1.051	0.834*	0.913	0.916
*CC is occurring*, *caused by human activities*	0.962	0.974	0.801**	0.714***	0.847*
Farmers’ adaptation attitude					
*Individual adaptation attitude*: *Farmers should change their production practices in the household*:	1.780***	1.344***	1.300***	1.255***	1.283***
*Climate change is a government job*, *not mine*	0.955	1.027	1.222**	1.062	0.930
Education	1.033*	0.953***	0.985	1.016	1.004
Age	0.992	0.997	1.003	0.999	0.985
Gender	1.114	1.066	0.949	1.995	0.896
Access services	1.209	0.844	0.845	0.739	0.793**
Access credit	0.949	0.905	0.820	0.951	1.039
Overall Model Statistics	*Pseudo R*^*2*^ *= 0*.*0413*	*Pseudo R*^*2*^ *= 0*.*0453*	*Pseudo R*^*2*^ *= 0*.*0367*	*Pseudo R*^*2*^ *= 0*.*0339*	*Pseudo R*^*2*^ *= 0*.*0403*

*** p<0.01,

** p<0.05,

* p<0.1.

Our results further showed that perceived risk from climatic threats is in a positive association (showing the value p < 0.001) with implementation of each adaptation approach. Climatic variation belief variable was found to be insignificant in correlation to diversifying plants and animals’ adaptation, climate change belief is caused by human activities was found be in significant association to implementation measures to adjust cultivation techniques, protect the land manage water usage. Education variable showed significant relationship with the diversifying plants and animals and applying new technology measures while increase in education levels leads to increase in likelihood of implementation to these measures. Farm households’ attitudes towards climate change adaptation variable was found to be in significant association with each strategy. Increase in farm households’ age variable showed significant relation with decreased farmer likelihood. Gender variable was found to be insignificant to adaptation strategies.

## 5. Discussion

Results of this study show that the most adaptive measure applied by farmers in the northern mountains is applying technology, followed by protect land and diversifying the animals whereas the results are similar to previous study conducted by Huong et al. [23]. However, most farmers only apply adaptation measures partially instead of apply as a short- and long-term management strategy. This is explained by limited capital, weak awareness of technology and extensive services of farmers [24, 25].

Increasing erosion and washing away soil are the risk that farmers are most worried and concerned, followed by the stress of prolonging the dry season and drought. This can be explained by changes in weather in the northern mountains of Vietnam recently. Northern mountains with steep slopes and low forest cover are easy to flash floods occurrences that may cause great loss of life and property [26]. Numerous studies have shown that flash floods are occurring more frequently with increasing severity [24]. Therefore, in order to improve farmers’ capacity to cope with climate change, the government needs to pay attention to flash flood adaptation strategies such as helping farmers to relocate to the safe places, build solid houses or increasing forest cover. The majority of farmers agree that they should change their production practices in the household. However, there is still a part of farmers who think that climate change is the Government’s support in implementing farmer’s adaptive measures. A change in the way to help farmers can be proactive to adapt climate change precautions.

Risk perception of northern mountainous farm households regarding climate change beliefs was found to be significant. Farmers who consider that climate change is affecting their lives had higher level of climate change perception [16, 27, 28]. Additionally, farmers who have higher level of perceived climatic risks to their farms had a higher belief in climate change especially due to human activities. This findings highlights the results that Le Dang et al. [13] studied the climate change risk perception of farm households in the Mekong Delta region of Vietnam. Our study established the critical role of risk perceptions due to climate change in the adaptation behaviors (as shown in Table 6) of farm households in northern mountainous area of Vietnam. The findings we have developed are consistent with A6rbuckle et al. [19] suggests that the moderate interactions are significantly linked with degree of confidence in anthropogenic climate risk and the perceived threats of climate change. In other words, farmers who agree that human actions contribute substantially to climate change are more careful and cautious about the effects than those who think it is only a “natural phenomenon”. These findings are also entirely consistent with H_2a_.

The key component of adaptation behaviors is the degree of concern for farmers with challenges such as heavy precipitation, drought, pests and diseases. This research emphasizes on the role of perception of climate risks among farmers in northern mountains of Vietnam (as illustrated in Tables 6 and 7). The findings indicate that it might be the first step towards mitigatory or adaptive behaviors to perceive the evidence of impacts. Our results are in agreement with the studies of Mase et al. [15] and Higginbotham et al. [11]. Perception of weather-related risk plays a significant part in farm households’ preference towards incorporation of weather-related decisions. Our findings are in line with the previous studies of [25], in which the researchers stated that farmers’ risk assessment and risk perceptions about flooding and heavy rain have been essential considerations for agricultural investment, development and risk management decisions. The researchers also suggested that farm households’ perception about risk and mindset need to be treated as core issues for the decision on risk coping strategies for agricultural sectors. Localized coordination and the preparation and leveling-up of multi-stakeholder action from local to regional scales are recommended to draw on the expectations of farmers of risk and confidence in weather fluctuations and associated coping capacity [23, 29].

## 6. Conclusion and policy implications

This study provides understanding of Vietnamese farmers response to climatic variations, perception of risks, and adaptation attitude towards climatic risks. In this study farmers who are living in mountainous area of Vietnam were targeted. Results of our study revealed that farmers in the northern mountains of Vietnam have applied several risk coping strategies to cope with risks and adaptive climate changes. Majority of farmers in the study area showed awareness of climatic variation and adaptation strategies that they implement partially instead of implementing them as a short-term or long-term risk management strategies. In this study, we used ordinary least squares regression for investigation of farmers’ belief in climatic variations, perception of anthropogenic climatic change, and risk attitude towards climatic hazards. Findings of the study showed that farmers in the study area were practicing risk coping strategies such as applying new technologies, adjustment of the seasonal calendar, and diversification strategy. The results further revealed that farmers in the study area were concerned about the risk in soil erosion and washout, followed by the stress of prolonged dry season and droughts. Similar to previous literature, our study has also some limitations, such as farmers’ attitudes and behaviors were measured at one point through cross-sectional survey, and ability to make inference reasons was limited. In addition, in survey responses, we analyzed and reported adaptive behaviors instead of observations. Our findings exposed the need of in-depth qualitative and quantitative research to understand how farmers think about climate change and adaptation strategies?. There is a need to study the willingness to pay for climate change adaptation activities and its influencing factors and to investigate that why farmers do not consider insurance program as a long-term risk management strategy as well as the risk attitude toward proactive technology improvement that affects adaptive behavior? Climatic events have serious implications for farm households’ livelihood bases in northern mountains of Vietnam. Consequently, it is imperative to improve a complete climate change adaptation approach minimizing vulnerability and to enhance the adaptive capacity of farm households. Our study findings further suggest that, due to climatic variations in the study area both off-farm and on-farm adaptations approaches need to contemplate local settings to advance suitable and ecological intrusions. Access to credit services, skill development training and guidance, is also critical for coping with climatic risks and indecision. Based on the extensive analytical analysis the research suggests useful implications for policymakers, economists and governmental agencies.

## Supporting information

S1 Dataset (XLS)

S1 File Questionnaire in English. (DOCX)

S2 File Questionnaire in Vietnamese. (DOCX)
