# Supplementary material for: Assessment of farm households’ perception, beliefs and attitude toward climatic risks: A case study of rural Vietnam
Source: PLoS One. 2021 Dec 28;16(12):e0258598. doi: 10.1371/journal.pone.0258598 (PMC8714103; doi:10.1371/journal.pone.0258598)
Supplement: S1 File — (DOCX) [file pone.0258598.s002.docx]

**HOUSEHOLD SURVEY**

**B. AWARENESS ABOUT CLIMATE CHANGE SITUATION IN THE LOCAL**

B1 . Have you ever heard about existence of climate change?

yes □ no □

B2 . If Yes, do you think annual temperature changing?

Increase □ Decrease □ Don't know □

B3 . Have you ever heard of greenhouse gas emissions?

Yes □ No □

B4 . If Yes do you think that there is a relationship between the application of fertilizers and greenhouse gas emissions?

Yes □ No □ Don't know □

B5 . Do you think that the intensive use of fertilizers and pesticides affects the quality of your local water source?

Yes □ No □ Don't know □

B6 . How do you think the temperature have changed in the last 10 years?

Increase □ Decrease □

No change □ Don't know □

B7 . How do you think the rainfall has changed in the last 10 years?

Increase □ Decrease □

No change □ Don't know □

B8 . How do you think that the number of droughts changes in the last 10 years?

Increase □ Decrease □

No change □ Don't know □

B9 . How do you think that the frequency of floods and landslides have changed in the last 10 years?

Increase □ Decrease □ No change □ Don't know □

B10 . We want to hear from you about the **causes of climate change** . Pleased confirm: agree or disagree with the statement after what extent (c more numbers from 1 to 5 (1 is totally disagree ... 5 is completely agree)

(Investor circle the **number** in the column of household responses)

| Causes of climate change | Totally disagree | Disagree | Slightly agree | Agree | Totally agree |
| --- | --- | --- | --- | --- | --- |
| There is not enough evidence to know whether climate change is happening or not | 1 | 2 | 3 | 4 | 5 |
| Climate change is happening and is mainly due to the change of natural environment | 1 | 2 | 3 | 4 | 5 |
| Climate change is happening and is mainly caused by human activities | 1 | 2 | 3 | 4 | 5 |
| Climate change is happening and the reason is due to both changes of nature and human activities | 1 | 2 | 3 | 4 | 5 |
| The emergence of many factories and industrial parks contributes to climate change | 1 | 2 | 3 | 4 | 5 |
| Population growth contributes to climate change | 1 | 2 | 3 | 4 | 5 |
| Urbanization (many cities, towns ...) causes climate change | 1 | 2 | 3 | 4 | 5 |
| Poor management of resources (land, forests, water ...) causes climate change | 1 | 2 | 3 | 4 | 5 |

B11. Among all possible climate change events that you have seen, please indicate the severity of the phenomenon that you still remember most by choosing one of the numbers from 1 to 5 .

| Phenomena | Absolutely no damage | Light damage | Moderate damage | Significant damage | Very serious damage |
| --- | --- | --- | --- | --- | --- |
| Heavy rain, whirlwind | 1 | 2 | 3 | 4 | 5 |
| Flood | 1 | 2 | 3 | 4 | 5 |
| Landslide | 1 | 2 | 3 | 4 | 5 |
| Prolonged drought | 1 | 2 | 3 | 4 | 5 |
| Rising temperature | 1 | 2 | 3 | 4 | 5 |
| Blizzard, icy winter with snow | 1 | 2 | 3 | 4 | 5 |
| Erratic rainy season | 1 | 2 | 3 | 4 | 5 |
| Other events (specify if any) ………………… | 1 | 2 | 3 | 4 | 5 |

B1 2 . Would you please give your opinion about the consequences of climate change in your area by indicating how much you agree or disagree with the following questions?

(Investor circle the **number** in the column of household responses)

| Phenomena | Totally disagree | Disagree | Slightly agree | Agree | Totally agree |
| --- | --- | --- | --- | --- | --- |
| Flash floods, heavy rains, thunderstorms ... caused damage to people and property | 1 | 2 | 3 | 4 | 5 |
| Prolonged drought affects crop yields | 1 | 2 | 3 | 4 | 5 |
| Rising temperatures cause more diseases for humans, crops and livestock | 1 | 2 | 3 | 4 | 5 |
| Irregular rainfall reduces crop yields | 1 | 2 | 3 | 4 | 5 |
| The temperature drops too low in the winter, killing plants and animals | 1 | 2 | 3 | 4 | 5 |

B13. In general, in your opinion, are the impacts of the above climate change phenomena increasing over the last 10 years?

1. Increase □ 2. Decrease □ 3. No change □ 4. I don't know □

B14. In the coming years, if climate change occurs and your family does not take any adaptive measures, how can your life be affected?

(The enumerator circles the selected **number** in the following column for the household answer)

| Affection | Never happen | Low Probability | Unsure | High probability of occurrence | Definitely happen |
| --- | --- | --- | --- | --- | --- |
| Affect health, illness, psychology | 1 | 2 | 3 | 4 | 5 |
| Affect to income | 1 | 2 | 3 | 4 | 5 |
| Affect assets (houses, land, property, machinery, furniture ...) | 1 | 2 | 3 | 4 | 5 |
| Affect yield and productivity of crops and livestock | 1 | 2 | 3 | 4 | 5 |
| Affect social relationships | 1 | 2 | 3 | 4 | 5 |
| Affect family relationships | 1 | 2 | 3 | 4 | 5 |

B15. In the coming years, if climate change occurs and your family does not take any adaptive measures, to what extent can your life be affected?

(The survey delineated tr on to **number** in the comments column of the household answer)

| Influence level | Absolutely no damage | Damage caused less | Moderate damage | Significant damage | Very serious damage |
| --- | --- | --- | --- | --- | --- |
| Affect health, disease, psychology | 1 | 2 | 3 | 4 | 5 |
| Affect income | 1 | 2 | 3 | 4 | 5 |
| Affect assets (houses, land, property, machinery, furniture ...) | 1 | 2 | 3 | 4 | 5 |
| Affect crop yield and productivity | 1 | 2 | 3 | 4 | 5 |
| Affect social relationships | 1 | 2 | 3 | 4 | 5 |
| Affect family relationships | 1 | 2 | 3 | 4 | 5 |

**C . DAMAGE OF HOUSEHOLDS**

C 1 . Does your household have members working far away (outside the commune, outside the district, province or oversea)?

Yes □ No □

C 2 . Did your household receive any government assistance (commune, district and province) in 1 the last year?

Yes □ No □

C 3 . Does your household have no current debt?

Yes □ No □

C 4 . Has none of members in your household got a chronic disease?

Yes □ No □

C 5 . Has none of members in your household got infectious diseases?

Yes □ No □

C 6 . Has any household member got sick leave?

Yes □ No □

C 7 . The average time from yourhousehold to the nearest health center is ................... minutes

C 8 . Daily food of yourfamily is mainly bought by:

from outside □ Food available from the family □

C 9 . Can your family be able to store any food during the year?

Yes □ No □

C 10 . In a year, do you have any months suffering from shortage in food supply?

Yes □ No □

C 11 . Water family activities you is taken primarily from:

Natural resources (Rivers,) □ Waterwork □

C 12 . In the dry season, do you have enough water for drinking?

Yes □ No □

C 13 . From house to the nearest water source you came to get water out for how long? ......... min

C 14 . Is your house solidly built as a constructed house rather than a contemporary house?

Yes □ No □

C 15. Has your house ever got damaged by extreme weather (eg storms, heavy rain, landslides cause subsidence ...) ?

Yes □ No □

C 16 . Have you met situation of lacking of cultivated land?

Yes □ No □

C 17 . In the past 10 years, how many flash floods have occurred in the locality? :

C 18 . In the past 10 years, how many landslides have the locality experienced? :

C 19 . In the past 10 years, how many droughts have the locality experienced? :

C 20 . Has your household have no members died by flash floods, landslides …?

Yes □ No □

C 21 . Has your household have no members injured by flash floods, landslides ... no?

Yes □ No □

C 22 . Were agricultural production or livestock/poultry damaged by flash floods, landslides, drought, snow?

Yes □ No □

C 23. If yes, how much was the damage estimated in terms of output (last year) ?

| Type of pet | Amount (number) | Weight (kg) |
| --- | --- | --- |
| Buffalo |  |  |
|  |  |  |
|  |  |  |
| Type tree |  |  |
| Paddy |  |  |

C 24 . If so, how much is the loss estimated? ( Million VND)

| Type of pet | Amount of money (million VND) |
| --- | --- |
| Buffalo |  |
|  |  |
|  |  |
| Type tree |  |
| Paddy |  |
|  |  |
|  |  |
|  |  |
|  |  |

**D . BELIEVING THE CLIMATE CHANGE AND ATTITUDE AT THE RISK**

D1 . Do you give your opinion on the following statements ?

| Targets | Totally disagree | Disagree | Slightly agree | Agree | Totally agree |
| --- | --- | --- | --- | --- | --- |
| Climate change is really happening | 1 | 2 | 3 | 4 | 5 |
| Dealing with climate change is responsible of government | 1 | 2 | 3 | 4 | 5 |
| I am worried about the potential impacts of climate change on my region's agriculture . | 1 | 2 | 3 | 4 | 5 |
| I am worried about the potential impacts of climate change on my household 's agricultural activities . | 1 | 2 | 3 | 4 | 5 |
| Changes in the weather are damaging my household's production | 1 | 2 | 3 | 4 | 5 |
| Changes in the weather are damaging my household's activities | 1 | 2 | 3 | 4 | 5 |
| I believe that extreme weather events will occur more often in the future | 1 | 2 | 3 | 4 | 5 |
| Climate change is not a big deal because human ingenuity will allow us to adapt to these changes. | 1 | 2 | 3 | 4 | 5 |

D2 . Are you worried about any potential extreme events that may affect your family's production activities?

| Threat | No worries | A little concerned | worried | Very concerned | Extremely worried |
| --- | --- | --- | --- | --- | --- |
| Increasing flash floods | 1 | 2 | 3 | 4 | 5 |
| Long dry season and drought | 1 | 2 | 3 | 4 | 5 |
| Increasing harmful insects | 1 | 2 | 3 | 4 | 5 |
| The high rate of plants disease | 1 | 2 | 3 | 4 | 5 |
| More frequent heavy rain | 1 | 2 | 3 | 4 | 5 |
| Increase the amount of landslide | 1 | 2 | 3 | 4 | 5 |
| Increase in extreme temperatures | 1 | 2 | 3 | 4 | 5 |
| Increasing erosion and washing away soil | 1 | 2 | 3 | 4 | 5 |

D 3 . If the temperature rises for a long time, with other conditions constant, do you change your crops and raising animals?

Yes □ No □

D 4. If yes, which plants and animals would you choose?

…………………………………………………………………………………...

D 5. If rainfall increases over a long period of time, with other conditions constant, do you change your plants and animals?

Yes □ No □

D 6 . If so, which plants and animals will you choose?

…………………………………………………………………………………...

D 7 . If rainfall decreases over a long period of time, with other conditions constant, do you change your plants and animals?

Yes □ No □

D 8 . If so, which plants and animals will you choose?

…………………………………………………………………………………...

**E . ASSESSMENT OF ADAPTATION TO CLIMATE CHANGE**

E1. Can your household have access to the agricultural and forestry extension services of the local?

Yes □ No □

E2. Can your household access to loans of any financial institution (bank, credit funds, funds to support farmers ...)?

Yes □ No □

E3. Has any of your family members joined political system?

Farmers Union □ Veterans Association □

Youth Union □ Women's Union □

Not participate □ other (................................) □

E4. Have your household suffered weather forecasts, severe weather alerts from any source ?

Yes □ No □

E5 . Do you give your opinion on the following statements ?

|  | Totally disagree | Disagree | Slightly agree | Agree | Totally agree |
| --- | --- | --- | --- | --- | --- |
| Farmers should apply soil protection measures due to the increase in rainfall | 1 | 2 | 3 | 4 | 5 |
| The government should increase investment in irrigation systems to cope with rainfall irregularities | 1 | 2 | 3 | 4 | 5 |
| The government should take measures to reduce emissions and the causes of climate change | 1 | 2 | 3 | 4 | 5 |

E6. Please tell us, what did your family do before climate change affected your production activities ?

| Adaptation strategy | Implemented as a long-term risk management strategy | Implemented as a short-term risk management strategy | Understanding and implementation | Knowing but applying partly | No implementation and no implementation plan |
| --- | --- | --- | --- | --- | --- |
| 1. Diversify plants and animals | 1 | 2 | 3 | 4 | 5 |
| + Raising more plants and animals | 1 | 2 | 3 | 4 | 5 |
| + Using more varieties of seeds and breeding | 1 | 2 | 3 | 4 | 5 |
| +Crop Rotation | 1 | 2 | 3 | 4 | 5 |
| 2. Application of new technology | 1 | 2 | 3 | 4 | 5 |
| + Using new varieties | 1 | 2 | 3 | 4 | 5 |
| + Applying new production techniques | 1 | 2 | 3 | 4 | 5 |
| 3. Adjusting the seasonal calendar | 1 | 2 | 3 | 4 | 5 |
| + Early sowing or harvesting | 1 | 2 | 3 | 4 | 5 |
| + Shorten season time | 1 | 2 | 3 | 4 | 5 |
| 4. Implementation of land protection measures (digging ditches, afforestation, reducing the use of pesticides, etc.) | 1 | 2 | 3 | 4 | 5 |
| 5. Adjustment of planting techniques | 1 | 2 | 3 | 4 | 5 |
| + Change the time of fertilizing, spraying | 1 | 2 | 3 | 4 | 5 |
| + Change irrigation time | 1 | 2 | 3 | 4 | 5 |
| 6. Managing water use | 1 | 2 | 3 | 4 | 5 |
| + Buying jars, building water tanks | 1 | 2 | 3 | 4 | 5 |
| + save water | 1 | 2 | 3 | 4 | 5 |
| + Reuse (like using vegetable washing water to water plants) | 1 | 2 | 3 | 4 | 5 |
| 7. Diversification of income | 1 | 2 | 3 | 4 | 5 |
| + Find more non-agricultural jobs | 1 | 2 | 3 | 4 | 5 |
| + Moving from agriculture to husbandry (partially or wholly) and vice versa | 1 | 2 | 3 | 4 | 5 |
| 8. Household financial management | 1 | 2 | 3 | 4 | 5 |
| + Enhance or expand current production scale | 1 | 2 | 3 | 4 | 5 |
| + Mobilizing capital to invest in new production | 1 | 2 | 3 | 4 | 5 |
| + Saving money | 1 | 2 | 3 | 4 | 5 |
| 9. Consolidating safety for people and property | 1 | 2 | 3 | 4 | 5 |
| + relocation or reinforcement of assets | 1 | 2 | 3 | 4 | 5 |
| + Planting forests or trees | 1 | 2 | 3 | 4 | 5 |
| + Seek or frequently listen to news about disaster forecasts | 1 | 2 | 3 | 4 | 5 |
| + Sell or rent a part of property | 1 | 2 | 3 | 4 | 5 |
| 10. Other measures | 1 | 2 | 3 | 4 | 5 |
| + Buy crop and livestock insurance | 1 | 2 | 3 | 4 | 5 |
| + Abandon farming jobs | 1 | 2 | 3 | 4 | 5 |

E7. You said the measures that our households have used to adapt, the effect to what extent?

| Adaptation strategy | Not implemented | Completely ineffective | Low efficiency | effective | Fairly effective | Highly effective |
| --- | --- | --- | --- | --- | --- | --- |
| 1. Diversify plants and animals | 0 | 1 | 2 | 3 | 4 | 5 |
| + Raising more plants and animals | 0 | 1 | 2 | 3 | 4 | 5 |
| + Using more varieties of seeds and breeding | 0 | 1 | 2 | 3 | 4 | 5 |
| +Crop Rotation | 0 | 1 | 2 | 3 | 4 | 5 |
| 2. Application of new technology | 0 | 1 | 2 | 3 | 4 | 5 |
| + Using new varieties | 0 | 1 | 2 | 3 | 4 | 5 |
| + Applying new production techniques | 0 | 1 | 2 | 3 | 4 | 5 |
| 3. Adjusting the seasonal calendar | 0 | 1 | 2 | 3 | 4 | 5 |
| + Early sowing or harvesting | 0 | 1 | 2 | 3 | 4 | 5 |
| + Shorten season time | 0 | 1 | 2 | 3 | 4 | 5 |
| 4. Implementation of land protection measures (digging ditches, afforestation, reducing the use of pesticides, etc.) | 0 | 1 | 2 | 3 | 4 | 5 |
| 5. Adjustment of planting techniques | 0 | 1 | 2 | 3 | 4 | 5 |
| + Change the time of fertilizing, spraying | 0 | 1 | 2 | 3 | 4 | 5 |
| + Change irrigation time | 0 | 1 | 2 | 3 | 4 | 5 |
| 6. Managing water use | 0 | 1 | 2 | 3 | 4 | 5 |
| + Buying jars, building water tanks | 0 | 1 | 2 | 3 | 4 | 5 |
| + save water | 0 | 1 | 2 | 3 | 4 | 5 |
| + Reuse (like using vegetable washing water to water plants) | 0 | 1 | 2 | 3 | 4 | 5 |
| 7. Diversification of income | 0 | 1 | 2 | 3 | 4 | 5 |
| + Find more non-agricultural jobs | 0 | 1 | 2 | 3 | 4 | 5 |
| + Moving from agriculture to husbandry (partially or wholly) and vice versa | 0 | 1 | 2 | 3 | 4 | 5 |
| 8. Household financial management | 0 | 1 | 2 | 3 | 4 | 5 |
| + Enhance or expand current production scale | 0 | 1 | 2 | 3 | 4 | 5 |
| + Mobilizing capital to invest in new production | 0 | 1 | 2 | 3 | 4 | 5 |
| + Saving money | 0 | 1 | 2 | 3 | 4 | 5 |
| 9. Consolidating safety for people and property | 0 | 1 | 2 | 3 | 4 | 5 |
| + relocation or reinforcement of assets | 0 | 1 | 2 | 3 | 4 | 5 |
| + Planting forests or trees | 0 | 1 | 2 | 3 | 4 | 5 |
| + Seek or frequently listen to news about disaster forecasts | 0 | 1 | 2 | 3 | 4 | 5 |
| + Sell or rent a part of property | 0 | 1 | 2 | 3 | 4 | 5 |
| 10. Other measures | 0 | 1 | 2 | 3 | 4 | 5 |
| + Buy crop and livestock insurance | 0 | 1 | 2 | 3 | 4 | 5 |
| + Abandon farming jobs | 0 | 1 | 2 | 3 | 4 | 5 |

E8. According to you, if you use these adaptation measures, how do they generally cost (including time, money and effort)?

| Adaptation strategy | Not implemented | no cost | Low | Neutral | Fairly | Extremely |
| --- | --- | --- | --- | --- | --- | --- |
| 1. Diversify plants and animals | 0 | 1 | 2 | 3 | 4 | 5 |
| + Raising more plants and animals | 0 | 1 | 2 | 3 | 4 | 5 |
| + Using more varieties of seeds and breeding | 0 | 1 | 2 | 3 | 4 | 5 |
| +Crop Rotation | 0 | 1 | 2 | 3 | 4 | 5 |
| 2. Application of new technology | 0 | 1 | 2 | 3 | 4 | 5 |
| + Using new varieties | 0 | 1 | 2 | 3 | 4 | 5 |
| + Applying new production techniques | 0 | 1 | 2 | 3 | 4 | 5 |
| 3. Adjusting the seasonal calendar | 0 | 1 | 2 | 3 | 4 | 5 |
| + Early sowing or harvesting | 0 | 1 | 2 | 3 | 4 | 5 |
| + Shorten season time | 0 | 1 | 2 | 3 | 4 | 5 |
| 4. Implementation of land protection measures (digging ditches, afforestation, reducing the use of pesticides, etc.) | 0 | 1 | 2 | 3 | 4 | 5 |
| 5. Adjustment of planting techniques | 0 | 1 | 2 | 3 | 4 | 5 |
| + Change the time of fertilizing, spraying | 0 | 1 | 2 | 3 | 4 | 5 |
| + Change irrigation time | 0 | 1 | 2 | 3 | 4 | 5 |
| 6. Managing water use | 0 | 1 | 2 | 3 | 4 | 5 |
| + Buying jars, building water tanks | 0 | 1 | 2 | 3 | 4 | 5 |
| + save water | 0 | 1 | 2 | 3 | 4 | 5 |
| + Reuse (like using vegetable washing water to water plants) | 0 | 1 | 2 | 3 | 4 | 5 |
| 7. Diversification of income | 0 | 1 | 2 | 3 | 4 | 5 |
| + Find more non-agricultural jobs | 0 | 1 | 2 | 3 | 4 | 5 |
| + Moving from agriculture to husbandry (partially or wholly) and vice versa | 0 | 1 | 2 | 3 | 4 | 5 |
| 8. Household financial management | 0 | 1 | 2 | 3 | 4 | 5 |
| + Enhance or expand current production scale | 0 | 1 | 2 | 3 | 4 | 5 |
| + Mobilizing capital to invest in new production | 0 | 1 | 2 | 3 | 4 | 5 |
| + Saving money | 0 | 1 | 2 | 3 | 4 | 5 |
| 9. Consolidating safety for people and property | 0 | 1 | 2 | 3 | 4 | 5 |
| + relocation or reinforcement of assets | 0 | 1 | 2 | 3 | 4 | 5 |
| + Planting forests or trees | 0 | 1 | 2 | 3 | 4 | 5 |
| + Seek or frequently listen to news about disaster forecasts | 0 | 1 | 2 | 3 | 4 | 5 |
| + Sell or rent a part of property | 0 | 1 | 2 | 3 | 4 | 5 |
| 10. Other measures | 0 | 1 | 2 | 3 | 4 | 5 |
| + Buy crop and livestock insurance | 0 | 1 | 2 | 3 | 4 | 5 |
| + Abandon farming jobs | 0 | 1 | 2 | 3 | 4 | 5 |

D9. Would you please tell me if the following adaptation measures have been taken by the state and the authorities in your area? If so, please let us know the benefits of these adaptation measures?

| Adaptation measures by local authorities | yes | No | No beneficial | Low | Moderate | Fair | Great |
| --- | --- | --- | --- | --- | --- | --- | --- |
| Propagate on television, radio and newspapers to warn about natural disasters and change the weather |  |  | 1 | 2 | 3 | 4 | 5 |
| Training in disaster prevention and rescue |  |  | 1 | 2 | 3 | 4 | 5 |
| Make plans to prevent flash floods and landslides |  |  | 1 | 2 | 3 | 4 | 5 |
| Building, strengthening embankments, embankments, dams |  |  | 1 | 2 | 3 | 4 | 5 |
| Construction of irrigation works, pumping stations |  |  | 1 | 2 | 3 | 4 | 5 |
| Bringing new varieties (drought tolerance, cold tolerance) to localities and encouraging farmers to use |  |  | 1 | 2 | 3 | 4 | 5 |
| Mobilize people to change crops to suit the weather conditions |  |  | 1 | 2 | 3 | 4 | 5 |
| Supporting varieties, finance and techniques |  |  | 1 | 2 | 3 | 4 | 5 |
| Develop and disseminate to farmers appropriate seasonal calendars |  |  | 1 | 2 | 3 | 4 | 5 |
| Enhance reforestation |  |  | 1 | 2 | 3 | 4 | 5 |
| Other measures (specify) ………………  ……………………… .. |  |  | 1 | 2 | 3 | 4 | 5 |

D10. Could you please give your confidence in the adaptation measures of local authorities?

| Criteria for assessing trust | Totally disagree | Disagree | Slightly agree | Agree | Totally agree |
| --- | --- | --- | --- | --- | --- |
| Local authorities know what to do to adapt to climate change | 1 | 2 | 3 | 4 | 5 |
| The adaptation measures of local authorities are implemented very timely | 1 | 2 | 3 | 4 | 5 |
| The adaptation measures of local authorities are very effective | 1 | 2 | 3 | 4 | 5 |
| The local weather and climate forecasting system works very well | 1 | 2 | 3 | 4 | 5 |
| Timely handled natural disasters by local authorities | 1 | 2 | 3 | 4 | 5 |

D11. Within 5 years, how will the estimated income change under the impact of climate change?

5 years ago [] Increase [] Decrease [] Unchanged

5 years later [] Increase [] Decrease [] Unchanged

- Full name of the interviewer
- GPS:
- Address:
- Age:
- Gender:
- Your marital status: [] Married [ ] Not married
- education (years):
- What is your family's religion
- [] Not following any religion [] Don't know [] Buddhism [] Christianity [] No answer

**A . GENERAL INFORMATION ABOUT HOUSEHOLD**

A1. how many people are living in the family:

A1.1. How many children in the family are under 15:

A2. Economic level of your household (according to local ranking)

[] Rich [] Fair [] Moderate [] Near Poor [] Poor

A3. Please provide your family income from the following sources:

| Income | Annual amount (million dong) |
| --- | --- |
| Cropping (after deducting the cost) |  |
| Livestock (after deducting costs) |  |
| Fisheries (after deducting costs) |  |
| Forestry (after deducting costs) |  |
| Sided occupation (after deducting costs) |  |
| Hired jobs |  |
| Collecting natural products (collecting medicinal plants, non-timber forest products, wild animals, etc.) |  |
| Subsidized |  |
| Pension |  |
| Salaries of civil servants |  |

A4. How many years has your household involved in agriculture?

A 5. How many people in your family have non- agricultural jobs?

A 6 . Production area of ​​the family

| Type | total area  (m ^2^ ) | family  (m ^2^ ) | Tenant | | Lease | | Actual cultivated area |
| --- | --- | --- | --- | --- | --- | --- | --- |
|  |  |  | Area (m ^2^ ) | cost (million VND) | Area (m ^2^ ) | Proceeds (million VND) |  |
| planning |  |  |  |  |  |  |  |
| Livestock |  |  |  |  |  |  |  |
| Forest |  |  |  |  |  |  |  |
| - Natural forests (protection) |  |  |  |  |  |  |  |
| - Production forests |  |  |  |  |  |  |  |
| Other land |  |  |  |  |  |  |  |

A 7 . The planted areas of main crop where your family works mostly on (ha /labor / m ^2^ ) -

…………………………………………………………

A 8 . What type of livestock and poultry do you have? How many?

………………………………………………………..

A 9 . The average distance from house to your farms (kilometers)? :

A10. The distance from house to the communal center (kilometers)? :

A11. The distance from house to the district center (kilometers)?

A1 2 . The distance from your house to the communal market (kilometers)?? :

A13. The distance from house to the medical center of the commune (kilometers)?:

A14. The distance from house to the medical center of the district (kilometers)?

A15. Any difficulty in transportation:

[] Only motor bike [] Only by foot [] Car can go []

A1 6 . Types of family properties?

| Property type | Unit | Amount | Value (million dong) |
| --- | --- | --- | --- |
| House |  |  |  |
| - Permanent house | m2 |  |  |
| - Semi-permanent house | m2 |  |  |
| - Temporary house | m2 |  |  |
| Livestock stall | m2 |  |  |
| Tractor | pcs |  |  |
| Plucking machine | pcs |  |  |
| Spraying machine | pcs |  |  |
| Motorcycle | pcs |  |  |
| Bike | pcs |  |  |
| Electric fan | pcs |  |  |
| Car | pcs |  |  |
| Water pumps | pcs |  |  |
| Television | pcs |  |  |
| Generator | pcs |  |  |
| Tillage machines | pcs |  |  |
| Washing machine | pcs |  |  |
| Fridge | pcs |  |  |
| Gas stove | pcs |  |  |
| Other…………………. |  |  |  |
|  |  |  |  |

|  |  |
| --- | --- |
