# Supplementary material for: Assessment of farm households’ perception, beliefs and attitude toward climatic risks: A case study of rural Vietnam
Source: PLoS One. 2021 Dec 28;16(12):e0258598. doi: 10.1371/journal.pone.0258598 (PMC8714103; doi:10.1371/journal.pone.0258598)
Supplement: S2 File — (DOCX) [file pone.0258598.s003.docx]

**PHIẾU ĐIỀU TRA HỘ GIA ĐÌNH**

Xin chào ông/bà! Chúng tôi đang tiến hành một nghiên cứu về nhận thức và hành vi thích ứng của người dân liên quan đến biến đổi khí hậu. Sự tham gia của ông/bà sẽ giúp cho chúng tôi rất nhiều và chúng tôi cảm ơn và đánh giá cao ý kiến của ông/bà.

Cảm ơn ông/bà đã đồng ý trả lời phỏng vấn!

Đầu tiên, chúng tôi muốn giải thích qua với ông/bà về biến đổi khí hậu. Biến đổi khí hậu là bất kỳ sự thay đổi nào về khí hậu trong một khoảng thời gian dài như hàng chục năm. Những biểu hiện của biến đổi khí hậu mà chúng ta thường thấy như xuất hiện mưa sớm hơn hay muộn hơn, lượng mưa tăng lên hay nhiều lên, nhiệt độ tăng hay giảm đi, các thiên tai thường xuyên hơn (như lũ quét, mưa to, sạt lở đất, hạn hán, bão…)

Chúng tôi xin phép tiếp tục phỏng vấn.

# **B. NHẬN THỨC VỀ TÌNH HÌNH BIẾN ĐỔI KHÍ HẬU TẠI ĐỊA PHƯƠNG**

B1. Ông bà đã bao giờ nghe nói về biến đổi khí hậu chưa?

Đã từng nghe □ Chưa nghe □

B2. Nếu Có, Sự thay đổi về nhiệt độ mà ông/bà nghe được đang tăng hay giảm dần đi?

Tăng dần □ Giảm dần □ Không biết □

B3. Ông/bà đã bao giờ nghe về khí thải nhà kính chưa?

Có □ Chưa nghe □

B4. Nếu Có, ông/bà có nghĩ rằng có một mối quan hệ giữa việc áp dụng phân bón và phát thải khí nhà kính?

Có □ Không □ Không biết □

B5. Ông/bà có nghĩ rằng việc sử dụng nhiều phân bón, thuốc trừ sâu có ảnh hưởng đến chất lượng nguồn nước của địa phương mình?

Có □ Không □ Không biết □

B6. Ông/bà nghĩ rằng nhiệt độ thay đổi như thế nào trong 10 năm vừa qua?

Tăng lên □ Giảm đi □

Không thay đổi □ Không biết □

B7. Ông/bà nghĩ rằng lượng mưa thay đổi như thế nào trong 10 năm vừa qua?

Tăng lên □ Giảm đi □

Không thay đổi □ Không biết □

B8. Ông/bà cho rằng hạn hán thay đổi như thế nào trong 10 năm vừa qua?

Tăng lên □ Giảm đi □

Không thay đổi □ Không biết □

B9. Ông/bà cho rằng lũ lụt, sạt lở đất thay đổi như thế nào trong 10 năm vừa qua?

Tăng lên □ Giảm đi □

Không thay đổi □ Không biết □

B10. Chúng tôi muốn biết ý kiến của ông/bà về những **nguyên nhân gây ra biến đổi khí hậu**. Vui lòng cho biết mình đồng ý hay không đồng ý với những câu nói sau ở mức độ nào. Vui lòng chọn trong các số từ 1 (hoàn toàn không đồng ý) đến 5 (hoàn toàn đồng ý) trên thẻ để diễn tả đúng nhất ý kiến của mình.

| Nguyên nhân biến đổi khí hậu | Hoàn toàn không đồng ý | Không đồng ý | Hình như vậy | Đồng ý | Hoàn toàn đồng ý |
| --- | --- | --- | --- | --- | --- |
| Không có đủ bằng chứng để biết biến đổi khí hậu có xảy ra hay không | 1 | 2 | 3 | 4 | 5 |
| Biến đổi khí hậu đang xảy ra và nguyên nhân chủ yếu do sự thay đổi của tự nhiên môi trường | 1 | 2 | 3 | 4 | 5 |
| Biến đổi khí hậu đang xảy ra và nguyên nhân chủ yếu do hoạt động của con người | 1 | 2 | 3 | 4 | 5 |
| Biến đổi khí hậu đang xảy ra và nguyên nhân là do cả sự thay đổi của tự nhiên và hoạt động của con người | 1 | 2 | 3 | 4 | 5 |
| Việc xuất hiện nhiều nhà máy, khu công nghiệp góp phần gây ra biến đổi khí hậu | 1 | 2 | 3 | 4 | 5 |
| Sự gia tăng dân số góp phần gây ra biến đổi khí hậu | 1 | 2 | 3 | 4 | 5 |
| Đô thị hóa (nhiều thành phố, thị trấn…) gây ra BĐKH | 1 | 2 | 3 | 4 | 5 |
| Việc quản lý tài nguyên (đất, rừng, nước…) không tốt gây ra biến đổi khí hậu | 1 | 2 | 3 | 4 | 5 |

B11. Trong những hiện tượng biến đổi khí hậu mà ông/bà đã thấy, vui lòng cho biết mức độ nghiêm trọng của hiện tượng mà mình còn nhớ nhất bằng cách chọn một trong các số từ 1 (hoàn toàn không thiệt hại) đến 5 (thiệt hại hết sức nghiêm trọng) trên thẻ để diễn tả đúng nhất ý kiến của mình

| Hiện tượng | Hoàn toàn không thiệt hại | Thiệt hại nhẹ | Thiệt hại vừa phải | Khá thiệt hại | Thiệt hại hết sức nghiêm trọng |
| --- | --- | --- | --- | --- | --- |
| Mưa lớn, mưa to, mưa lâu, gió lốc | 1 | 2 | 3 | 4 | 5 |
| Lũ lụt | 1 | 2 | 3 | 4 | 5 |
| Sạt lở đất | 1 | 2 | 3 | 4 | 5 |
| Hạn hán kéo dài | 1 | 2 | 3 | 4 | 5 |
| Nắng nóng nhiều hơn | 1 | 2 | 3 | 4 | 5 |
| Nhiệt độ quá thấp, có băng, có tuyết | 1 | 2 | 3 | 4 | 5 |
| Mưa trái mùa, thất thường | 1 | 2 | 3 | 4 | 5 |
| Hiện tượng khác (ghi rõ nếu có)………………… | 1 | 2 | 3 | 4 | 5 |

B12. Ông bà vui lòng cho biết ý kiến về hậu quả của biến đổi khí hậu nơi mình sống bằng cách cho biết mình đồng ý hay không đồng ý với những câu hỏi sau ở mức độ nảo?

| Hiện tượng | Hoàn toàn không đồng ý | Không đồng ý | Hình như vậy | Đồng ý | Hoàn toàn đồng ý |
| --- | --- | --- | --- | --- | --- |
| Lũ quét, mưa lớn, giông bão… gây thiệt hại cho con người và tài sản | 1 | 2 | 3 | 4 | 5 |
| Hạn hán kéo dài ảnh hưởng đến sản lượng cây trồng | 1 | 2 | 3 | 4 | 5 |
| Nhiệt độ tăng cao gây ra nhiều dịch bệnh hơn cho con người và cây trồng, vật nuôi | 1 | 2 | 3 | 4 | 5 |
| Mưa thất thường làm giảm năng suất cây trồng | 1 | 2 | 3 | 4 | 5 |
| Nhiệt độ giảm quá thấp vào mùa đông làm chết cây trồng và vật nuôi | 1 | 2 | 3 | 4 | 5 |

B13. Nhìn chung, theo ông/bà tác hại của những hiện tượng biến đổi khí hậu ở trên trong khoảng thời gian 10 năm qua tăng, giảm hay không đổi?

1. Tăng □ 2. Giảm □

3. Không thay đổi □ 4. Tôi không biết □

B14. Trong những năm tới, nếu biến đổi khí hậu xảy ra mà gia đình ông/bà không có biện pháp thích ứng nào thì đời sống của hộ gia đình ông/bà có thể bị ảnh hưởng như thế nào?

| Ảnh hưởng | Không bao giờ xảy ra | Khả năng xảy ra thấp | Có thể có hoặc không | Khả năng xảy ra cao | Chắc chắn sẽ xảy ra |
| --- | --- | --- | --- | --- | --- |
| Ảnh hưởng đến sức khỏe, bệnh tật, tâm lý | 1 | 2 | 3 | 4 | 5 |
| Ảnh hưởng đến thu nhập | 1 | 2 | 3 | 4 | 5 |
| Ảnh hưởng đến tài sản (nhà cửa, đất đai, tài sản, máy móc, đồ đạc…) | 1 | 2 | 3 | 4 | 5 |
| Ảnh hưởng đến sản lượng và năng suất cây trồng vật nuôi | 1 | 2 | 3 | 4 | 5 |
| Ảnh hưởng đến các mối quan hệ xã hội | 1 | 2 | 3 | 4 | 5 |
| Ảnh hưởng đến các mối quan hệ gia đình | 1 | 2 | 3 | 4 | 5 |

B15. Trong những năm tới, nếu biến đổi khí hậu xảy ra mà gia đình ông/bà không có biện pháp thích ứng nào thì đời sống của hộ gia đình ông/bà có thể bị ảnh hưởng ở mức độ nào?

| Mức độ ảnh hưởng | Hoàn toàn không thiệt hại | Thiaạt hại ít | Thiệt hại vừa phải | Thiệt hại khá nhiều | Thiệt hại hết sức nghiêm trọng |
| --- | --- | --- | --- | --- | --- |
| Ảnh hưởng đến sức khỏe, bệnh tật, tâm lý | 1 | 2 | 3 | 4 | 5 |
| Ảnh hưởng đến thu nhập | 1 | 2 | 3 | 4 | 5 |
| Ảnh hưởng đến tài sản (nhà cửa, đất đai, tài sản, máy móc, đồ đạc…) | 1 | 2 | 3 | 4 | 5 |
| Ảnh hưởng đến sản lượng và năng suất cây trồng vật nuôi | 1 | 2 | 3 | 4 | 5 |
| Ảnh hưởng đến các mối quan hệ xã hội | 1 | 2 | 3 | 4 | 5 |
| Ảnh hưởng đến các mối quan hệ gia đình | 1 | 2 | 3 | 4 | 5 |

# **C. TỔN THƯƠNG CỦA HỘ GIA ĐÌNH**

C1. Hộ gia đình ông/bà có thành viên đi làm ăn xa (bên ngoài xã, ngoài huyện, tỉnh hoặc nước ngoài) không?

Có □ Không □

C2. Hộ gia đình của ông/bà có nhận bất kỳ sự trợ giúp nào của chính quyền (xã, huyện, tỉnh) trong 1 năm vừa qua không?

Có □ Không □

C3. Hộ gia đình của ông/bà có khoản vay nợ nào hiện nay không?

Có □ Không □

C4. Có thành viên nào trong hộ gia đình ông/bà bị mắc bệnh mãn tính không?

Có □ Không □

C5. Có thành viên nào trong hộ gia đình ông/bà bị mắc bệnh truyền nhiễm không?

Có □ Không □

C6. Có thành viên nào trong hộ gia đình ông/bà phải nghỉ học, hoặc nghỉ việc do bị ốm trong 1 năm vừa qua không?

Có □ Không □

C7. Thời gian trung bình từ hộ ông/bà đến trung tâm y tế gần nhất là ................... phút

C8. Thực phẩm hàng ngày của gia đình ông/bà chủ yếu là:

Mua từ bên ngoài □ Thực phẩm có sẵn của gia đình □

C9. Gia đình ông/bà có tích trữ lương thực trong năm không?

Có □ Không □

C10. Trong một năm, gia đình ông/bà có mấy tháng không đủ lương thực sử dụng?

Có □ Không □

C11. Nước sinh hoạt của gia đình ông/bà được lấy chủ yếu từ:

Nguồn tự nhiên (Sông, hồ, suối) □ Nước máy □

C12. Vào mùa khô, gia đình ông/bà có đủ nước cho sinh hoạt không?

Có □ Không □

C13. Từ gia đình ông/bà đến chỗ lấy nước hết bao lâu?............................ phút

C14. Nhà của ông/bà đang ở có được xây dựng kiên cố không?

Có □ Không □

C15. Nhà của ông/bà có bao giờ bị ảnh hưởng, bị hư hỏng do thời tiết cực đoan (ví dụ: bão, mưa to, sạt lở gây lún…) không?

Có □ Không □

C16. Hộ gia đình của ông/bà có bị thiếu đất canh tác không?

Có □ Không □

C17. Trong 10 năm vừa qua địa phương có xảy ra bao nhiêu trận lũ quét?:

C18. Trong 10 năm vừa qua địa phương có xảy ra bao nhiêu trận sạt lở đất?:

C19. Trong 10 năm vừa qua địa phương có xảy ra bao nhiêu đợt hạn hán?:

C20. Hộ gia đình ông/bà có thành viên nào bị thiệt mạng do lũ quét, sạt lở đất… không?

Có □ Không □

C21. Hộ gia đình ông/bà có thành viên nào bị thương do lũ quét, sạt lở đất… không?

Có □ Không □

# **D. NIỀM TIN VÀO BIẾN ĐỔI KHÍ HẬU VÀ THÁI ĐỘ ĐỐI VỚI RỦI RO**

D1. Ông/bà cho biết ý kiến của mình về những phát biểu sau?

| Chỉ tiêu | Hoàn toàn kô đồng ý | Không đồng ý | Hình như vậy | Đồng ý | Hoàn toàn đồng ý |
| --- | --- | --- | --- | --- | --- |
| Biến đổi khí hậu thực sự đang diễn ra | 1 | 2 | 3 | 4 | 5 |
| Biến đổi khí hậu là việc của chính quyền chứ không phải của tôi | 1 | 2 | 3 | 4 | 5 |
| Tôi lo lắng về những tác động tiềm tàng của biến đổi khí hậu đối với nông nghiệp của cả vùng. | 1 | 2 | 3 | 4 | 5 |
| Tôi lo lắng về những tác động tiềm tàng của biến đổi khí hậu đối với hoạt động nông nghiệp của hộ gia đình tôi. | 1 | 2 | 3 | 4 | 5 |
| Những thay đổi trong thời tiết đang làm tổn hại đến hoạt động sản xuất của hộ gia đình tôi | 1 | 2 | 3 | 4 | 5 |
| Những thay đổi trong thời tiết đang làm tổn hại đến sinh hoạt của hộ gia đình tôi | 1 | 2 | 3 | 4 | 5 |
| Tôi tin rằng các sự kiện thời tiết khắc nghiệt sẽ xảy ra thường xuyên hơn trong tương lai | 1 | 2 | 3 | 4 | 5 |
| Biến đổi khí hậu không phải là một vấn đề lớn bởi vì sự khéo léo của con người sẽ cho phép chúng ta thích nghi với những thay đổi | 1 | 2 | 3 | 4 | 5 |

D2. Ông/bà lo ngại về các hiện tượng cực đoan tiềm tàng nào có thể ảnh hưởng cho hoạt động sản xuất của gia đình?

| Mối đe dọa | Không lo ngại | Lo ngại một chút | Lo ngại | Rất lo ngại | Cực kì lo ngại |
| --- | --- | --- | --- | --- | --- |
| Gia tăng lũ quét | 1 | 2 | 3 | 4 | 5 |
| Mùa khô kéo dài và hạn hán | 1 | 2 | 3 | 4 | 5 |
| Gia tăng côn trùng gây hại | 1 | 2 | 3 | 4 | 5 |
| Tỷ lệ cây trồng bị bệnh cao | 1 | 2 | 3 | 4 | 5 |
| Mưa cực đoan thường xuyên hơn | 1 | 2 | 3 | 4 | 5 |
| Gia tăng lượng đất đai bị mất | 1 | 2 | 3 | 4 | 5 |
| Gia tăng nhiệt độ cực đoan | 1 | 2 | 3 | 4 | 5 |
| Gia tăng xói mòn và rửa trôi đất | 1 | 2 | 3 | 4 | 5 |

D3. Nếu nhiệt độ tăng lên trong thời gian dài, với các diều kiện khác không đổi, ông/bà có chuyển đổi cây trồng, vật nuôi hay không?

Có □ Không □

D4. Nếu có thì ông/bà sẽ lựa chọn loại cây trồng, vật nuôi nào?

D5. Nếu lượng mưa tăng lên trong thời gian dài, với các diều kiện khác không đổi, ông/bà có chuyển đổi cây trồng, vật nuôi hay không?

Có □ Không □

D6. Nếu có thì ông/bà sẽ lựa chọn loại cây trồng, vật nuôi nào?

D7. Nếu lượng mưa giảm đi trong thời gian dài, với các diều kiện khác không đổi, ông/bà có chuyển đổi cây trồng, vật nuôi hay không?

Có □ Không □

D8. Nếu có thì ông/bà sẽ lựa chọn loại cây trồng, vật nuôi nào?

# **E. ĐÁNH GIÁTHÍCH ỨNG VỚI BIẾN ĐỔI KHÍ HẬU**

D1. Hộ gia đình ông/bà có được tiếp cận với các dịch vụ khuyến nông khuyến lâm của địa phương không?

Có □ Không □

D2. Hộ gia đình của ông/bà có tiếp cận được các khoản vốn vay của bất kỳ tổ chức tài chính (ngân hàng, quỹ tín dụng, quỹ hỗ trợ nông dân…) không?

Có □ Không □

D3. Hộ gia đình của ông/bà có thành viên tham gia tổ chức chính trị, xã hội nào của địa phương ?

Hội nông dân □ Hội cựu chiến binh □

Đoàn thanh niên □ Hội phụ nữ □

Không tham gia □ Khác (................................) □

D4. Hộ gia đình ông/bà có nhận được thông tin dự báo thời tiết, cảnh báo thời tiết nguy hiểm từ bất kỳ nguồn nào không?

Có □ Không □

D5. Xin Ông/bà cho biết ý kiến về các phát biểu sau:

|  | Rất không đồng ý | Không đồng ý | Không chắc chắn | Đồng ý | Rất đồng ý |
| --- | --- | --- | --- | --- | --- |
| Nông dân nên áp dụng các biện pháp bảo vệ đất do sự gia tăng của lượng mưa | 1 | 2 | 3 | 4 | 5 |
| Chính phủ nên tăng cường đầu tư vào hệ thống thủy lợi để đối phó với sự thất thường của mưa | 1 | 2 | 3 | 4 | 5 |
| Chính phủ nên áp dụng các biện pháp giảm phát thải và các nguyên nhân gây biến đổi khí hậu | 1 | 2 | 3 | 4 | 5 |

D6. Xin Ông/Bà cho biết, trước tình trạng biến đổi khí hậu ảnh hưởng đến hoạt động sản xuất, gia đình Ông/Bà đã làm gì?

| Chiến lược thích ứng | Thực hiện như một chiến lược quản lý rủi ro dài hạn hoặc ngắn hạn | Không thực hiện nhưng quan tâm | Không thực hiện và không có kế hoạch thực hiện |
| --- | --- | --- | --- |
| 1. Đa dạng hóa cây trồng, vật nuôi | 1 | 2 | 3 |
| + Gieo trồng nhiều loại cây, nuôi nhiều loại vật nuôi | 1 | 2 | 3 |
| + Sử dụng nhiều loại giống | 1 | 2 | 3 |
| + Luân canh | 1 | 2 | 3 |
| 2. Áp dụng công nghệ mới | 1 | 2 | 3 |
| + Sử dụng giống mới | 1 | 2 | 3 |
| + Áp dụng kỹ thuật sản xuất mới | 1 | 2 | 3 |
| 3. Điều chỉnh lịch thời vụ | 1 | 2 | 3 |
| + Xuống giống/gieo sạ hoặc thu hoạch sớm | 1 | 2 | 3 |
| + Rút ngắn thời gian của 1 vụ | 1 | 2 | 3 |
| 4. Thực hiện các biện pháp bảo vệ đất (đào rãnh, mương, trồng rừng, giảm sử dụng thuốc trừ sâu…) | 1 | 2 | 3 |
| 5. Điều chỉnh kỹ thuật gieo trồng | 1 | 2 | 3 |
| + Thay đổi thời gian bón phân, phun thuốc | 1 | 2 | 3 |
| + Thay đổi thời gian tưới tiêu | 1 | 2 | 3 |
| 6. Quản lý việc sử dụng nước | 1 | 2 | 3 |
| + Mua lu, xây bể chứa nước | 1 | 2 | 3 |
| + Sử dụng nước tiết kiệm | 1 | 2 | 3 |
| + Tái sử dụng (như dùng nước rửa rau để tưới cây) | 1 | 2 | 3 |
| 7. Đa dạng hóa nguồn thu nhập | 1 | 2 | 3 |
| + Tìm thêm việc làm phi nông nghiệp khác | 1 | 2 | 3 |
| + Chuyển từ trồng trọt sang chăn nuôi (1 phần hay toàn bộ) và ngược lại | 1 | 2 | 3 |
| 8. Quản lý tài chính hộ | 1 | 2 | 3 |
| + Tăng cường hoặc mở rộng quy mô sản xuất hiện tại | 1 | 2 | 3 |
| + Huy động vốn để đầu tư sản xuất mới | 1 | 2 | 3 |
| + Gửi tiết kiệm | 1 | 2 | 3 |
| 9. Củng cố an toàn cho người và tài sản | 1 | 2 | 3 |
| + di dời hoặc gia cố tài sản | 1 | 2 | 3 |
| + Trồng rừng hoặc cây gỗ | 1 | 2 | 3 |
| + Xem hoặc nghe các tin tức dự báo về thiên tai | 1 | 2 | 3 |
| + Bán hoặc cho thuê một phần tài sản | 1 | 2 | 3 |
| 10. Biện pháp khác | 1 | 2 | 3 |
| + Mua bảo hiểm cây trồng, vật nuôi | 1 | 2 | 3 |
| + Thoát khỏi ngành, từ bỏ nông nghiệp | 1 | 2 | 3 |

D7. Ông/bà cho biết những biện pháp mà hộ gia đình ta đã sử dụng để thích ứng thì hiệu quả mang lại ở mức độ nào?

| Chiến lược thích ứng | Hoàn toàn không có hiệu quả | Hiệu quả thấp | Có hiệu quả vừa phải | Có hiệu quả khá | Có hiệu quả cao |
| --- | --- | --- | --- | --- | --- |
| 1. Đa dạng hóa cây trồng, vật nuôi | 1 | 2 | 3 | 4 | 5 |
| + Gieo trồng nhiều loại cây, nuôi nhiều loại vật nuôi | 1 | 2 | 3 | 4 | 5 |
| + Sử dụng nhiều loại giống | 1 | 2 | 3 | 4 | 5 |
| + Luân canh | 1 | 2 | 3 | 4 | 5 |
| 2. Áp dụng công nghệ mới | 1 | 2 | 3 | 4 | 5 |
| + Sử dụng giống mới | 1 | 2 | 3 | 4 | 5 |
| + Áp dụng kỹ thuật sản xuất mới | 1 | 2 | 3 | 4 | 5 |
| 3. Điều chỉnh lịch thời vụ | 1 | 2 | 3 | 4 | 5 |
| + Xuống giống/gieo sạ hoặc thu hoạch sớm | 1 | 2 | 3 | 4 | 5 |
| + Rút ngắn thời gian của 1 vụ | 1 | 2 | 3 | 4 | 5 |
| 4. Thực hiện các biện pháp bảo vệ đất (đào rãnh, mương, trồng rừng, giảm sử dụng thuốc trừ sâu…) | 1 | 2 | 3 | 4 | 5 |
| 5. Điều chỉnh kỹ thuật gieo trồng | 1 | 2 | 3 | 4 | 5 |
| + Thay đổi thời gian bón phân, phun thuốc | 1 | 2 | 3 | 4 | 5 |
| + Thay đổi thời gian tưới tiêu | 1 | 2 | 3 | 4 | 5 |
| 6. Quản lý việc sử dụng nước | 1 | 2 | 3 | 4 | 5 |
| + Mua lu, xây bể chứa nước | 1 | 2 | 3 | 4 | 5 |
| + Sử dụng nước tiết kiệm | 1 | 2 | 3 | 4 | 5 |
| + Tái sử dụng (như dùng nước rửa rau để tưới cây) | 1 | 2 | 3 | 4 | 5 |
| 7. Đa dạng hóa nguồn thu nhập | 1 | 2 | 3 | 4 | 5 |
| + Tìm thêm việc làm phi nông nghiệp khác | 1 | 2 | 3 | 4 | 5 |
| + Chuyển từ trồng trọt sang chăn nuôi (1 phần hay toàn bộ) và ngược lại | 1 | 2 | 3 | 4 | 5 |
| 8. Quản lý tài chính hộ | 1 | 2 | 3 | 4 | 5 |
| + Tăng cường hoặc mở rộng quy mô sản xuất hiện tại | 1 | 2 | 3 | 4 | 5 |
| + Huy động vốn để đầu tư sản xuất mới | 1 | 2 | 3 | 4 | 5 |
| + Gửi tiết kiệm | 1 | 2 | 3 | 4 | 5 |
| 9. Củng cố an toàn cho người và tài sản | 1 | 2 | 3 | 4 | 5 |
| + di dời hoặc gia cố tài sản | 1 | 2 | 3 | 4 | 5 |
| + Trồng rừng hoặc cây gỗ | 1 | 2 | 3 | 4 | 5 |
| + Xem hoặc nghe các tin tức dự báo về thiên tai | 1 | 2 | 3 | 4 | 5 |
| + Bán hoặc cho thuê một phần tài sản | 1 | 2 | 3 | 4 | 5 |
| 10. Biện pháp khác | 1 | 2 | 3 | 4 | 5 |
| + Mua bảo hiểm cây trồng, vật nuôi | 1 | 2 | 3 | 4 | 5 |
| + Thoát khỏi ngành, từ bỏ nông nghiệp | 1 | 2 | 3 | 4 | 5 |

D8. Theo ông/bà, nếu sử dụng những biện pháp thích ứng này, nhìn chung cần chi phí như thế nào (bao gồm cả thời gian, tiền bạc và công sức)

| Chiến lược thích ứng | Hoàn toàn không có chi phí gì | Chi phí ít | Chi phí vừa phải | Chi phí khá | Chi phí vô cùng lớn |
| --- | --- | --- | --- | --- | --- |
| 1. Đa dạng hóa cây trồng, vật nuôi | 1 | 2 | 3 | 4 | 5 |
| + Gieo trồng nhiều loại cây, nuôi nhiều loại vật nuôi | 1 | 2 | 3 | 4 | 5 |
| + Sử dụng nhiều loại giống | 1 | 2 | 3 | 4 | 5 |
| + Luân canh | 1 | 2 | 3 | 4 | 5 |
| 2. Áp dụng công nghệ mới | 1 | 2 | 3 | 4 | 5 |
| + Sử dụng giống mới | 1 | 2 | 3 | 4 | 5 |
| + Áp dụng kỹ thuật sản xuất mới | 1 | 2 | 3 | 4 | 5 |
| 3. Điều chỉnh lịch thời vụ | 1 | 2 | 3 | 4 | 5 |
| + Xuống giống/gieo sạ hoặc thu hoạch sớm | 1 | 2 | 3 | 4 | 5 |
| + Rút ngắn thời gian của 1 vụ | 1 | 2 | 3 | 4 | 5 |
| 4. Thực hiện các biện pháp bảo vệ đất (đào rãnh, mương, trồng rừng, giảm sử dụng thuốc trừ sâu…) | 1 | 2 | 3 | 4 | 5 |
| 5. Điều chỉnh kỹ thuật gieo trồng | 1 | 2 | 3 | 4 | 5 |
| + Thay đổi thời gian bón phân, phun thuốc | 1 | 2 | 3 | 4 | 5 |
| + Thay đổi thời gian tưới tiêu | 1 | 2 | 3 | 4 | 5 |
| 6. Quản lý việc sử dụng nước | 1 | 2 | 3 | 4 | 5 |
| + Mua lu, xây bể chứa nước | 1 | 2 | 3 | 4 | 5 |
| + Sử dụng nước tiết kiệm | 1 | 2 | 3 | 4 | 5 |
| + Tái sử dụng (như dùng nước rửa rau để tưới cây) | 1 | 2 | 3 | 4 | 5 |
| 7. Đa dạng hóa nguồn thu nhập | 1 | 2 | 3 | 4 | 5 |
| + Tìm thêm việc làm phi nông nghiệp khác | 1 | 2 | 3 | 4 | 5 |
| + Chuyển từ trồng trọt sang chăn nuôi (1 phần hay toàn bộ) và ngược lại | 1 | 2 | 3 | 4 | 5 |
| 8. Quản lý tài chính hộ | 1 | 2 | 3 | 4 | 5 |
| + Tăng cường hoặc mở rộng quy mô sản xuất hiện tại | 1 | 2 | 3 | 4 | 5 |
| + Huy động vốn để đầu tư sản xuất mới | 1 | 2 | 3 | 4 | 5 |
| + Gửi tiết kiệm | 1 | 2 | 3 | 4 | 5 |
| 9. Củng cố an toàn cho người và tài sản | 1 | 2 | 3 | 4 | 5 |
| + di dời hoặc gia cố tài sản | 1 | 2 | 3 | 4 | 5 |
| + Trồng rừng hoặc cây gỗ | 1 | 2 | 3 | 4 | 5 |
| + Xem hoặc nghe các tin tức dự báo về thiên tai | 1 | 2 | 3 | 4 | 5 |
| + Bán hoặc cho thuê một phần tài sản | 1 | 2 | 3 | 4 | 5 |
| 10. Biện pháp khác | 1 | 2 | 3 | 4 | 5 |
| + Mua bảo hiểm cây trồng, vật nuôi | 1 | 2 | 3 | 4 | 5 |
| + Thoát khỏi ngành, từ bỏ nông nghiệp | 1 | 2 | 3 | 4 | 5 |

D9. Ông/bà vui lòng cho biết những biện pháp thích ứng sau đây đã được nhà nước và chính quyền thực hiện ở dịa phương mình chưa? Nếu đã thực hiện vui lòng cho chúng tôi biết ích lợi của các biện pháp thích ứng này ở mức độ nào?

| Các biện pháp thích ứng của chính quyền địa phương | Có | Kô | Hoàn toàn không có ích lợi gì | Ích lợi ít | Ích lợi vừa vừa | Khá Có ích lợi khá | Ích lợi rất lớn |
| --- | --- | --- | --- | --- | --- | --- | --- |
| Tuyên truyền trên tivi, báo đài cảnh báo về thiên tai, thay đổi thời tiết |  |  | 1 | 2 | 3 | 4 | 5 |
| Tập huấn phòng chống thiên tai và cứu hộ cứu nạn |  |  | 1 | 2 | 3 | 4 | 5 |
| Lập kế hoạch phòng chống lũ quét và sạt lở đất |  |  | 1 | 2 | 3 | 4 | 5 |
| Xây dựng, gia cố bờ kè, đê bao, cống đập vững chắc |  |  | 1 | 2 | 3 | 4 | 5 |
| Xây dựng các công trình thủy lợi, trạm bơm |  |  | 1 | 2 | 3 | 4 | 5 |
| Đưa giống mới (chịu hạn, chịu rét) về địa phương và khuyến khích nông dân sử dụng |  |  | 1 | 2 | 3 | 4 | 5 |
| Vận động bà con chuyển đổi cây trồng cho phù hợp với điều kiện thời tiết |  |  | 1 | 2 | 3 | 4 | 5 |
| Hỗ trợ giống, vốn, kỹ thuật |  |  | 1 | 2 | 3 | 4 | 5 |
| Xây dựng và phổ biến cho nông dân lịch thời vụ phù hợp |  |  | 1 | 2 | 3 | 4 | 5 |
| Tăng cường trồng rừng |  |  | 1 | 2 | 3 | 4 | 5 |
| Biện pháp khác (ghi rõ)………………  ……………………….. |  |  | 1 | 2 | 3 | 4 | 5 |

D10. Xin ông/bà cho biết sự tin tưởng của ông/bà vào các biện pháp thích ứng của chính quyền địa phương?

| Các chỉ tiêu đánh giá sự tin tưởng | Hoàn toàn không đồng ý | Không đồng ý | Hình như vậy | Đồng ý | Hoàn toàn đồng ý |
| --- | --- | --- | --- | --- | --- |
| Chính quyền địa phương biết phải làm gì để thích ứng với biến đổi khí hậu | 1 | 2 | 3 | 4 | 5 |
| Các biện pháp thích ứng của chính quyền địa phương được thực hiện rất kịp thời | 1 | 2 | 3 | 4 | 5 |
| Các biện pháp thích ứng của chính quyền địa phương được thực hiện rất hiệu quả | 1 | 2 | 3 | 4 | 5 |
| Hệ thống cảnh báo về thời tiết, khí hậu của địa phương hoạt động rất tốt | 1 | 2 | 3 | 4 | 5 |
| Việc xử lý sau thiên tai của chính quyền địa phương kịp thời | 1 | 2 | 3 | 4 | 5 |

Họ và tên người được phỏng vấn:

GPS:

Địa chỉ:

Tuổi: Giới tính:

Tình trạng hôn nhân của ông/bà: [ ] Đã lập gia đình [ ] Chưa lập gia đình

Ông/bà đã học hết lớp mấy:

Gia đình Ông/bà theo đạo gì (câu hỏi này tế nhị, cần quan sát khu vực xung quanh, có nhà thờ hay chùa gì không hoặc quan sát cách bài trí cách thờ cúng trong gia đình):

[ ] Không theo đạo gì [ ] Không biết [ ] Đạo phật [ ] Thiên chúa giáo [ ] Không trả lời

Số lần đi lễ trong năm:

# **A. THÔNG TIN CHUNG VỀ HỘ GIA ĐÌNH**

A1. Gia đình hiện nay có bao nhiêu người đang sinh sống:

A1.1. Gia đình có bao nhiêu trẻ em dưới 15:

A2. Mức độ kinh tế của hộ (theo xếp loại của địa phương)

[ ] Giàu [ ] Khá [ ] Trung bình [ ] Cận nghèo [ ] Nghèo

A3. Ông/bà vui lòng cho biết thu nhập của gia đình mình từ các nguồn sau:

| Nguồn thu nhập | Số tiền hàng năm (triệu đồng) |
| --- | --- |
| Trồng trọt (sau khi trừ chi phí) |  |
| Chăn nuôi (sau khi trừ chi phí) |  |
| Thủy sản (sau khi trừ chi phí) |  |
| Lâm nghiệp (sau khi trừ chi phí) |  |
| Ngành nghề phụ (sau khi trừ chi phí) |  |
| Làm thuê |  |
| Hái lượm các sản phẩm tự nhiên (thu hái cây thuốc, lâm sản ngoài gỗ, săn bắt cá tự nhiên…) |  |
| Trợ cấp |  |
| Lương hưu |  |
| Lương công chức, viên chức |  |

A4. Hộ gia đình ông/bà đã làm nông nghiệp được bao nhiêu năm?

A5. Trong gia đình có bao nhiêu người làm nghề khác ngoài nông nghiệp?

A6. Diện tích đất sản xuất của gia đình

| Lọai đât | Tổng diện tích  (m^2^) | Của gia đình  (m^2^) | Đi thuê | | Cho thuê | | Diện tích canh tác thực tế |
| --- | --- | --- | --- | --- | --- | --- | --- |
|  |  |  | D.tích (m^2^) | Chi phí thuê (tr.đ) | D.tích (m^2^) | Tiền thu được (tr.đ) |  |
| Đất trồng trọt |  |  |  |  |  |  |  |
| Đất chăn nuôi |  |  |  |  |  |  |  |
| Đất rừng |  |  |  |  |  |  |  |
| - Rừng tự nhiên (bảo vệ) |  |  |  |  |  |  |  |
| - Rừng sản xuất |  |  |  |  |  |  |  |
| Đất khác |  |  |  |  |  |  |  |

A7. Diện tích các cây trồng chính mà gia đình ông/bà có là bao nhiêu (ha/công/sào/m^2^/mẫu) - Ghi rõ tên cây trồng và diện tích:

1. 3.

2 4.

A8. Gia đình ông/bà có chăn nuôi loại gia súc, gia cầm nào? Số lượng bao nhiêu? - ghi rõ tên gia súc, gia cầm và số lượng con.

1 2

3 4

A9. Khoảng cách từ nhà ông\bà đến ruộng\nương khoảng bao nhiêu km?:

A10. Khoảng cách từ nhà ông\bà đến trung tâm xã khoảng bao nhiêu km?:

A11. Khoảng cách từ nhà ông\bà đến trung tâm huyện khoảng bao nhiêu km?:

A12. Khoảng cách từ ruộng\nương nhà ông\bà đến chợ trung tâm là bao nhiêu km?:

A13. Khoảng cách từ nhà ông\bà đến trung tâm y tế xã khoảng bao nhiêu km?:

A14. Khoảng cách từ nhà ông\bà đến trung tâm y tế huyện khoảng bao nhiêu km?:

A15. Giao thông thuận lợi hay khó khăn:

[ ] Đi xe máy dễ dàng [ ] Đi bộ [ ] Ô tô đi được [ ]

A16. Các loại tài sản của gia đình?

| Loại tài sản | ĐVT | Số lượng | Giá trị (triệu đồng) |
| --- | --- | --- | --- |
| Nhà |  |  |  |
| - Nhà kiên cố | m2 |  |  |
| - Nhà bán kiên cố | m2 |  |  |
| - Nhà tạm bợ | m2 |  |  |
| Chuồng trại chăn nuôi | m2 |  |  |
| Máy cày | chiếc |  |  |
| Máy tuốt lúa | chiếc |  |  |
| Bình phun thuốc sâu | chiếc |  |  |
| Xe máy | chiếc |  |  |
| Xe đạp | chiếc |  |  |
| Quạt điện | chiếc |  |  |
| Ô tô | chiếc |  |  |
| Máy bơm nước | chiếc |  |  |
| Ti vi | chiếc |  |  |
| Máy phát điện | chiếc |  |  |
| Máy làm đất | chiếc |  |  |
| Máy giặt | chiếc |  |  |
| Tủ lạnh | chiếc |  |  |
| Bếp ga | chiếc |  |  |
| Khác…………………. |  |  |  |
|  |  |  |  |

**Người điều tra**
